# Ezh2 mediated H3K27me3 activity facilitates somatic transition during human pluripotent reprogramming

**DOI:** 10.1038/srep08229

**Published:** 2015-02-04

**Authors:** Radhika Arasala Rao, Narendra Dhele, Sabna Cheemadan, Alhad Ketkar, Giridhara R. Jayandharan, Dasaradhi Palakodeti, Shravanti Rampalli

**Affiliations:** 1 Centre For Inflammation and Tissue Homeostasis, Institute for Stem Cell Biology and Regenerative Medicine (inStem), National Centre for Biological Sciences, GKVK Campus, Bellary Road, Bangalore 560065, Karnataka, India; 2 Sastra University, Tirumalaisamudram, Thanjavur - 613 401, TamilNadu, India; 3 Department of Hematology and Centre for Stem Cell Research, Christian Medical College, Vellore - 632004, TamilNadu, India

## Abstract

Factor induced reprogramming of fibroblasts is an orchestrated but inefficient process. At the epigenetic level, it results in drastic chromatin changes to erase the existing somatic “memory” and to establish the pluripotent state. Accordingly, alterations of chromatin regulators including Ezh2 influence iPSC generation. While the role of individual transcription factors in resetting the chromatin landscape during iPSC generation is increasingly evident, their engagement with chromatin modulators remains to be elucidated. In the current study, we demonstrate that histone methyl transferase activity of Ezh2 is required for mesenchymal to epithelial transition (MET) during human iPSC generation. We show that the H3K27me3 activity favors induction of pluripotency by transcriptionally targeting the TGF-β signaling pathway. We also demonstrate that the Ezh2 negatively regulates the expression of pro-EMT miRNA's such as miR-23a locus during MET. Unique association of Ezh2 with c-Myc was required to silence the aforementioned circuitry. Collectively, our findings provide a mechanistic understanding by which Ezh2 restricts the somatic programme during early phase of cellular reprogramming and establish the importance of Ezh2 dependent H3K27me3 activity in transcriptional and miRNA modulation during human iPSC generation.

Forced expression of the transcription factors Oct4, Sox2, Klf4 and c-Myc (OSKM) alter the fate of somatic cell to a pluripotent state^1^,^2^,^3^. These induced pluripotent cells iPSCs share molecular and functional features of embryonic stem cells ESCs and therefore hold great promise for understanding human development and disease^4^. Although a variety of somatic cell types can be reprogrammed, our current understanding of the molecular mechanisms and cellular nature of reprogramming is almost exclusively derived from fibroblasts^5^,^6^,^7^,^8^. At the chromatin level, reprogramming of fibroblasts is initiated by inhibition of somatic gene expression along with rapid acquisition of H3K4me2 on several promoters and enhancers of genes that are transcriptionally activated later during the reprogramming process^9^. This widespread remodeling of histone modifications acts as an immediate response and is consistent with the fact that the perturbation of somatic gene expression is a prerequisite for cellular reprogramming^10^. To accomplish such massive epigenomic changes, pluripotency transcription factors direct the recruitment of chromatin modulators to repress the fibroblast specific programme. In this regard, studies have documented that the deletion of repressive chromatin modulators such as Polycomb proteins (PcG), Ehmt1 and Ehmt2 inhibited iPSC generation while knockdown of their respective demethylases UTX, JmJD3 and JARID2c enhances the process^11^,^12^,^13^.

PcG proteins are comprised of multiprotein complexes PRC1 and PRC2 that are required for conveying cellular memory by transcriptional silencing of a subset of genes^14^. The PRC2 core is composed of the catalytic subunit Ezh2, non-enzymatic Suz12, and EED components that catalyzes the histone H3 methylation at lysine-27 residue^14^. Genome-wide binding of PRC2 in human and mouse pluripotent cells demonstrated binding overlap with pluripotency factors on the promoters of genes encoding developmental regulators that are required for lineage specification later during development^15^. Consistently, these genes are enriched for the domains containing repressive H3K27me3 and activating H3K4me3 that are deposited by polycomb (PcG) and trithorax (Trx) complexes, to hold the promoters of developmental regulators in a poised state^16^. The importance of PcG is underscored by the deletion of PRC2 components in mouse embryonic stem cells, which results in global de-repression of target genes^15^,^17^,^18^ followed by spontaneous differentiation^18^. Furthermore, genetic ablation of PRC2 components in mice results in developmental failures and early embryonic lethality^19^,^20^. Ezh2 also plays an important role in maintaining the identity of multipotent adult hematopoietic, neural and muscle precursors stem cells^21^,^22^. A recent report has demonstrated the increased expression of PRC2 components during mouse fibroblast reprogramming^23^. Moreover, knockdown of PRC2 components, including Ezh2, Suz12 and EED, have been shown to drastically reduce iPSC generation from human and mouse fibroblasts^12^,^23^,^24^,^25^. Besides, the requirement of PRC2 components in factor-induced reprogramming is supported by Pereira et al.'s (2010) observation^24^ wherein PRC2 deficient ESCs failed to reprogram differentiated cells to pluripotency in heterokaryon assays.

Given the importance of the PcG protein complex in resetting the epigenetic barrier, it is necessary to uncover the mechanism by which these regulators govern iPSC generation. Therefore, in the current study we investigated the requirement of Ezh2 and its methyltransferase activity in human iPSC generation using gain/loss of function approaches and by using specific small molecular inhibitor of H3K27 activity. We discovered Ezh2 and its H3K27me3 activity represses pro-EMT signaling during reprogramming. We also determined that Ezh2 along with c-Myc assist in somatic transition by silencing TGF-β signaling and the miR-23a locus. Our data provides a mechanistic understanding by which Ezh2 overcomes the initial impediments of cellular reprogramming.

## Results

### PRC2 components are induced during human fibroblasts reprogramming

**Figure 1:**
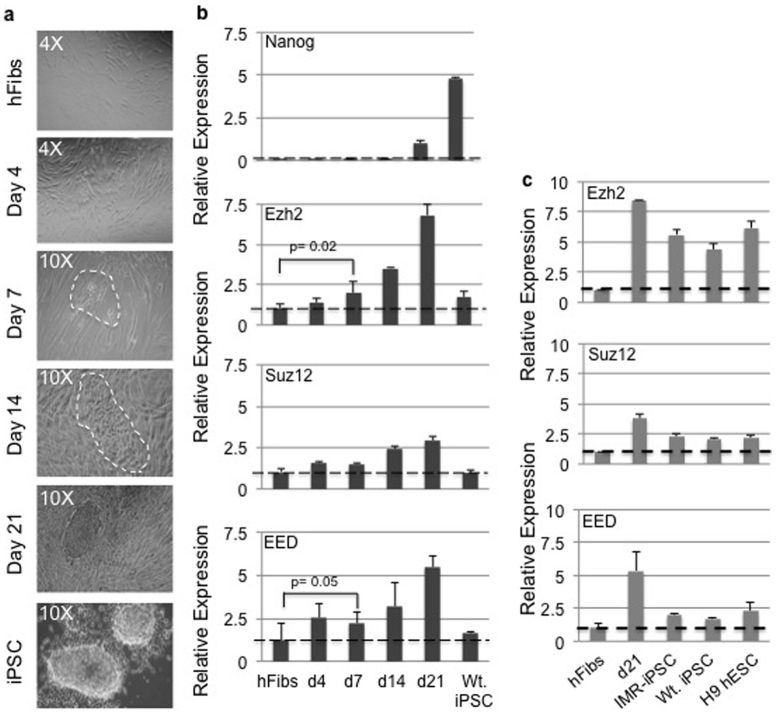
Enhanced expression of PRC2 components in human fibroblast reprogramming. (a) Bright field images of hFibs undergoing reprogramming upon OSKM transduction. Cells within the white dashed line at day 7 indicate the appearance of epithelial morphology in hFib cultures. These cells grew to make bigger colony structure by day 14, eventually generating the iPSC like colony at day 21. picking the colony allowed us to establish the iPSC line. (b) Expression profile of indicated mRNAs at various time courses of hFib reprogramming. (c) Expression profile of PRC2 components (Ezh2, Suz12 and Eed) in human fibroblasts, day 21 reprogramming cultures and various human pluripotent cells.

We first decided to verify the kinetics of PRC2 expression during human iPSC (hiPS) generation. Towards this hFibs (human fibroblasts) were transduced with OSKM and cultures were monitored for phenotypic changes by phase contrast microscopy along with molecular analysis. Consistent with previous reports^7^, clusters of cells with epithelial morphology indicative of mesenchymal to epithelial transition (MET) appeared at day 7, which eventually matured to generate iPS colonies by three weeks (Fig. 1a). These colonies were picked to establish iPSC lines. In addition, we assayed for Nanog expression, a key pluripotency marker required for the acquisition of complete reprogramming as positive control. We did not observe detectable Nanog transcripts in hFibs at day 4, 7 or 14. Nanog was expressed by day 21 and in an established iPSC line (Fig. 1b) demonstrating that our reprogramming timelines were in agreement with that reported by other groups. iPSC colonies generated from hFibs exhibited typical ESC morphology and expressed the cell surface marker Tra 1-60 well as the pluripotency transcription factors Oct4 and Nanog (Supplementary Fig. 1a–c). H9-hESC obtained from WiCell Research Institute were used as a positive control for Tra 1-60, Oct4 and Nanog staining.

Transcript profiling of polycomb members over the same time course indicated an increase in Suz12 and Eed expression as early as day 4 post OSKM introduction, while Ezh2 upregulation was noticed on day 7. Expression of all three polycomb members continued to increase until 21 days after addition of reprogramming factors (Fig. 1b). We also observed higher expression of Ezh2 in iPSCs compared to human fibroblasts (Fig. 1b). Ding et al^23^ showed highest expression of three PRC2 components in established miPS and mES lines when measured against MEFs and MEFs undergoing reprogramming. However unlike mouse pluripotent cells, established hiPS and hES lines showed reduced expression of PRC2 components in comparison with advanced time courses in the reprogramming assay (Day 21) (Fig. 1c). While there are no reports comparing the amount of Ezh2 in mouse and human pluripotent cells, the differences between mouse and human pluripotent cells could be attributed to the naïve vs. primed state and proliferation mode. Currently it is our speculation that Ezh2 expression is reduced once stable pluripotent lines are established from heterogeneous reprogramming cultures. Nonetheless, our studies revealed an increase in the kinetics of PRC2 expression during human fibroblasts reprogramming.

### Ezh2 mediated H3K27me3 activity is critical in the early phase of reprogramming

To evaluate the role of Ezh2 in human iPSC generation we used a loss of function approach in the reprogramming assays. Validated shRNAs targeting the ORF (shEzh2 74) and 3′ UTR (shEzh2 75) of Ezh2 (Supplementary Fig. 2a–c) were transduced in hFibs prior to OSKM transduction (schema shown in Supplementary Fig. 2d). Using three biological replicates and three experimental replicates we observed a 0.05% frequency of iPSC generation from Wt and shCnt hFibs (Supplementary Fig. 1e) and no colony formation in hFibs transduced with shEzh2-74 or shEzh2-75. Investigations into the molecular details of shEzh2-transduced cells demonstrated an increased expression of p53, p21, and p19 both at the transcript and protein levels (Supplementary Fig. 2f,g) along with slower growth rate and cellular senescence (Supplementary Fig. 2h). Accordingly, OSKM transduced shEzh2 hFibs cultures displayed flat morphology of cells, strong reduction in proliferation and noticeable cell death compared to OSKM transduced Wt and shCnt starting at day 4 (data not shown).

**Figure 2:**
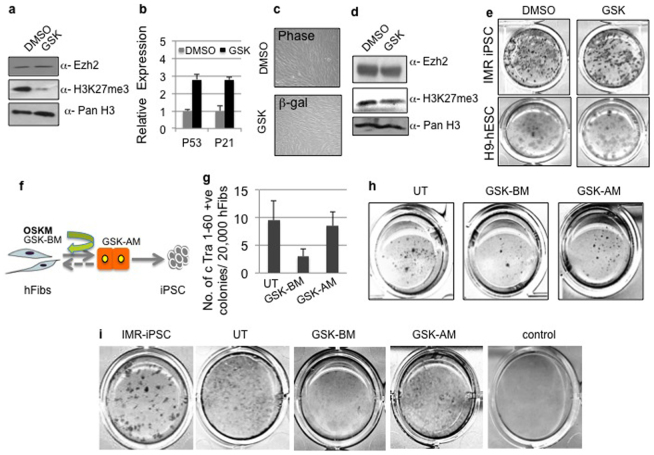
Inhibition of H3K27me3 activity impairs mesenchymal to epithelial transition during iPSC generation. (a) Western blot analysis using anti Ezh2, anti-H3K27Me3 and control anti-pan H3 antibodies in hFibs treated with H3K27me3 inhibitor GSK-126. Uncropped/Full blots images are provided in Supplementary Fig. 6 a-c. (b) Relative expression of p53 and p21 mRNA in DMSO control and GSK treated hFibs. (c) Representative bright field and β- gal staining images upon treatment of hFibs with DMSO and GSK-126. (d) Western blot analysis using anti Ezh2, anti-H3K27Me3 and control anti-pan H3 antibodies in H9 hESC treated with DMSO or H3K27me3 inhibitor GSK-126. Uncropped/Full blots images are provided in Supplementary Fig. 6 d–f. (e) Tra 1-60 DAB staining in IMR-90 iPSC, H9 hESC cultures treated with DMSO and GSK-126. (f) Schematic representation of reprogramming protocol and time points of addition of GSK to the reprogramming cultures. (g) Total number of reprogrammed colonies generated three weeks post OSKM transduced hFibs and upon GSK treatment at indicated time points in schema (h) Tra 1-60 DAB staining on day 21 post addition of reprogramming factors and GSK treatment (i) E-cad DAB staining on day 10 post addition of reprogramming factors and GSK treatment as indicated in schema.

While there are several reports indicating removal of Ezh2 from somatic cells leads to senescence by induction of p53, p21 and ARF proteins^26^,^27^, it is not clear if inhibition of its H3K27me3 activity would have the same senescence-inducing effect on hFibs. Thus we studied the effect of GSK-126 a small molecule inhibitor of Ezh2 on hFib cultures. Treatment of GSK126 inhibited H3K27me3 activity without altering Ezh2 protein levels in human fibroblasts (Fig. 2a). Similar to the effect of shEzh2, GSK increased the expression of p53 and p21, albeit at lower levels without affecting the cell shape or proliferation (Fig. 2b,c). Furthermore, GSK treated cells did not undergo cell senescence, which was detected by β-galactosidase staining (Fig. 2c). Collectively, these results suggest that the loss of Ezh2 versus abrogation of methyltransferase activity alone seems to have a differential effect on cell proliferation in somatic fibroblast cells. This was particularly surprising since transduction of shRNA or treatment with inhibitors ultimately reduced overall H3K27me3 levels.

Ezh2 mediated H3K27me3 activity is implicated in the maintenance of bivalent chromatin domains in pluripotent cells. In addition Ezh2 is abundantly expressed in both ESC and iPS cells. Therefore we determined the importance of Ezh2 and H3K27me3 activity in maintaining the pluripotent state. We transduced shEzh2 in Wt.iPSC and observed over 30% reduction of Ezh2 transcript (Supplementary Fig. 2i, j). Lowered levels of Ezh2 in iPSC had no appreciable difference in colony morphology and expression of pluripotency markers such as Tra 1-60 and Nanog (Supplementary Fig. 2j). Next we treated IMR iPSC and H9 hESC with DMSO or GSK. Similar to that seen in fibroblasts, GSK treatment reduced H3K27me3 methylation over 70% without influencing Ezh2 expression (Fig. 2d). Cultures were monitored for retaining the pluripotency marker Tra 1-60 at least three passages in presence of DMSO or GSK. Staining for pluripotency marker Tra 1-60 did not indicate loss of pluripotency in DMSO control and GSK treated hPSC cultures (IMR90 iPSC and H9 hESC) (Fig. 2e). Since GSK treatment did not affect hFib proliferation or pluripotency of hPSC we speculated that use of GSK in reprogramming assays would allow us to identify additional roles of H3K27me3 in iPSC generation.

Reprogramming experiments were broadly divided into two phases i.e before mesenchymal to epithelial transition (MET) and after MET. GSK was added to cultures as indicated in the schema (Fig. 2f). To our surprise the number of Tra 1-60 positive iPSC colonies generated were strongly reduced in pre- MET GSK treated cultures (Before MET-BM) compared to post MET (after MET-AM) (Fig. 2g,h). To further confirm whether the effect of GSK treatment is specific to mesenchymal to epithelial transition we repeated the experiment as indicated in Fig. 2f. Ten days post OSKM transduction we stained the reprogramming cultures with E-cadherin antibody. As shown in Fig. 2i the number of E-cadherin positive colonies in GSK-BM was drastically reduced compared to untreated controls. E-Cadherin staining in IMR-iPSC was used as the positive control in Fig. 2i. Together these results demonstrate that H3K27me3 activity plays an important role in facilitating the mesenchymal to epithelial transition during iPSC generation.

### Ezh2 mediated H3K27me3 activity inhibits Pro-EMT signaling

**Figure 3:**
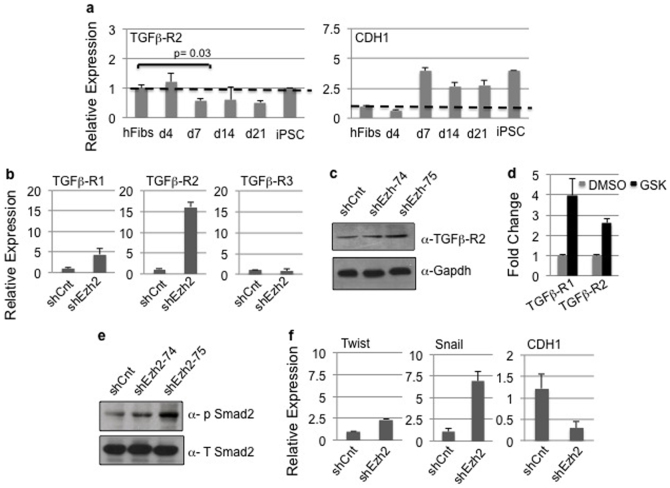
Inactivation of Ezh2 or inhibition of H3K27me3 activity enhances TGF-β signaling in human fibroblasts. (a) Expression profile of TGF-βR2 and CDH1 in hFibs undergoing reprogramming at various time courses indicated. (b) Expression profile of mRNAs encoding TGF-β receptors in hFibs transduced with control and Ezh2 shRNA. (c) Western blotting for TGF-βR2 and GAPDH control in hFibs transduced with control and Ezh2 shRNA. Uncropped/Full blots images are provided in Supplementary Fig. 7 a–b. (d) Expression profile of mRNAs encoding TGF-β receptors in DMSO and GSK treated human fibroblasts. (e) Western blotting using anti phospho- Smad2 and total Smad2 antibody in hFibs transduced with control and Ezh2 shRNA. Uncropped/Full blots images are provided in Supplementary Fig. 7 c–d (f) Relative expression of mRNA for Twist, Snail and E-cadherin (CDH1) that are downstream targets for TGF-β signaling in hFibs transduced with control and Ezh2 shRNA.

Overcoming the blockade of TGF-β signaling is a hallmark of MET and is critical for pluripotent reprogramming^7^,^28^,^29^. Accordingly, treatment of fibroblasts with TGF-β inhibitors improves iPSC formation^30^. Based on our results suggesting the important link between H3K27me3 activity and MET, we speculated that Ezh2 negatively regulate TGF-β signaling and thus suppresses EMT. Accordingly, among all the polycomb members, upregulation of Ezh2 coincided with diminished TGF-βR2 expression on day 7 of reprogramming (Fig. 3a left). We also observed overexpression of the epithelial marker E-cadherin (CDH1) coinciding with the appearance of epithelial cells on the 7^th^ day of reprogramming (Fig. 3a right). These results are in alignment with our findings where MET was reduced from GSK treated hFibs (Fig. 2g,i).

To validate the inverse correlation between Ezh2 and TGF-βR2 that was observed during reprogramming, we performed loss and gain of Ezh2 function in human fibroblasts. Towards this we experimentally verified expression of members of TGF-β and BMP pathway in shEzh2 hFibs. Compared to shCnt, we observed increased expression of TGF-β R1 and R2 at both the transcript and protein levels while TGF-β R3 or TGF-β ligands remain unchanged (Fig. 3b,c and Supplementary Fig. 3a). Results of Ezh2 knockdown were confirmed by inhibitor treatment wherein GSK treatment increased expression of TGF-β receptor expression (Fig. 3d). If differential expression of TGR-β receptors is directly linked to knockdown of Ezh2, and its H3K27me3 activity then overexpression should reverse this trend. To test this hypothesis, hFibs were transduced with retrovirus encoding Ezh2 transgene (Ezh2 OE). Molecular investigation at transcript and protein level identified increased Ezh2 expression associated with reversed levels of TGF-β receptors (Supplementary Fig. 3b–3c). In contrast to knockdown, Ezh2 overexpression decreased the expression of TGF-βR1 and R2 suggesting that these receptors are direct targets of Ezh2 mediated H3K27me3 activity (Supplementary Fig. 3d–3e).

Activation of the TGF-β pathway involves phosphorylation of Smads that ultimately activate the mesenchymal transcription factor Snail. Accordingly, we also detected phosphorylation of Smad2 (Fig. 3e) along with increased mRNA expression of Twist and Snail in sh-Ezh2 transduced hFibs (Fig. 3f). Expression of epithelial E-cadherin (CDH1) is indicative of MET initiation during the reprogramming process and is negatively correlated with TGF-β activation. We observed downregulation of E-cadherin (CDH1) in shEzh2 hFibs (Fig. 3f). These results confirm the Ezh2 depletion releases the repression on TGF-β receptors thereby activating downstream TGF-β signaling. Overall our results demonstrate that Ezh2 mediated H3K27me3 activity restricts TGF-β signaling thereby dictating the reprogramming outcome.

Unlike TGF-β signaling, components of the BMP pathway such as BMP receptor I/II synergize with OSKM to promote iPSC generation^29^. We observed reduced transcript levels of BMP receptor II and I in shEzh2 hFibs (Supplementary Fig. 3f). Taken together, our results demonstrate Ezh2 mediated H3K27me3 activity regulate fibroblast specific gene expression programme by restricting TGF-β signaling. Nonetheless, these results support our model that Ezh2 and its H3K27me3 promotes reprogramming by inhibiting EMT.

### Inhibition of TGF-β signaling rescues the H3K27me3 mediated initiation impairment during reprogramming

After establishing that H3K27me3 activity is required to suppress TGF-β signaling and thus pro-EMT signaling, we tested if inhibition of TGF-β signaling is sufficient to rescue the initiation defect from shEzh2 or GSK treated hFibs. To test this hypothesis, reprogramming experiments were performed in the presence or absence of TGF-β inhibitor SB431542 (SB) as indicated in the schema (Supplementary Fig. 4a). While control hFibs showed the appearance of E-cadherin positive colonies around day 7, shEzh2 hFibs or shEzh2 hFibs treated with SB did not show changes in-vitro (Supplementary Fig. 4b). Molecular analysis indicated that shEzh2 transduced hFibs continued to show reduced Ezh2 and increased TGF-βR1/R2 transcript irrespective of SB treatment (Supplementary Fig. 4c). Moreover we observed enhanced CDH1 mRNA in SB treated shEzh2 treated hFibs indicating that the programme associated with MET was set in place upon inhibition of TGF-β signaling (Supplementary Fig. 4c). Further probing into reasons for the lack of MET in shEzh2 treated with SB indicated transient recovery of cell senescence (Supplementary Fig. 4d) and eventual growth crises due to enhanced p21 levels (Supplementary Fig. 4e).

**Figure 4:**
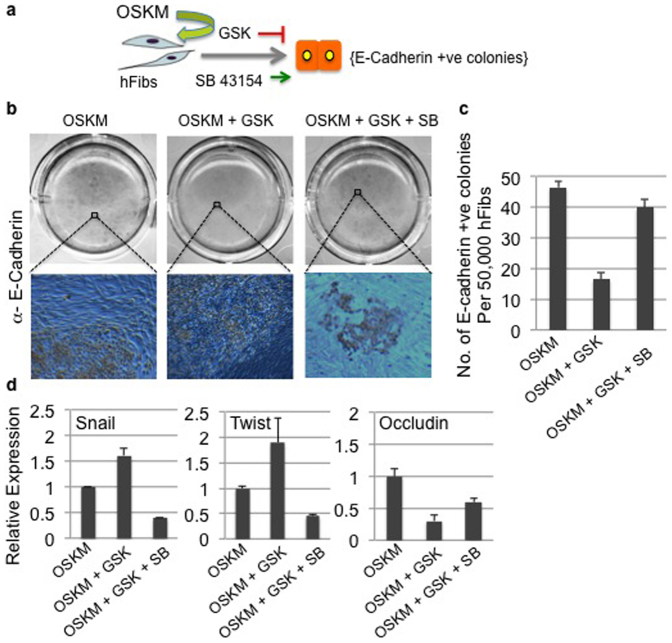
Inhibition of TGF-β signaling rescues the H3K27me3 mediated MET defect. (a) Schematic representation for OSKM transduction, GSK and SB treatment during hFib reprogramming. (b) E-Cadherin DAB staining of OSKM transduced cultures treated with indicated inhibitors. (c) Total number of E-cadherin positive colonies from OSKM, OSKM+GSK and OSKM+GSK+SB cultures. (d) Relative expression of indicated mRNAs from OSKM, OSKM+GSK and OSKM+GSK+SB reprogramming cultures.

Since we demonstrated removal of Ezh2 protein vs. inhibition of PRC2 activity has differential effects in terms of induction of cell senescence in human fibroblasts (Fig. 2 and Supplementary Fig. 2), we tested whether SB can reverse the impaired MET from GSK treated hFibs. Towards this we added SB and GSK to OSKM transduced cultures as indicated in the schema (Fig. 4a). Interestingly GSK + SB treated fibroblasts showed similar number of E-cadherin positive colonies as untreated hFibs (Fig. 4b,c), indicating that MET defects due to H3K27me3 inhibition could be rescued by inhibition of downstream TGF-β signaling. Molecular analysis of fibroblast specific markers such as Twist and Snail showed elevated expression upon GSK treatment and their levels went down in GSB+SB treated cells (Fig. 4d). In alignment with our rescue data upon SB treatment, we also observed elevated levels of epithelial maker Occludin in GSK+SB treated cultures (Fig. 4d). Cumulatively our studies have established that H3K27me3 activity is required to suppress the fibroblast specific programme setup by TGF-β signaling in order to facilitate a mesenchymal to epithelial transition.

### Ezh2 interacts with c-Myc to suppress TGF-β Signaling

**Figure 5:**
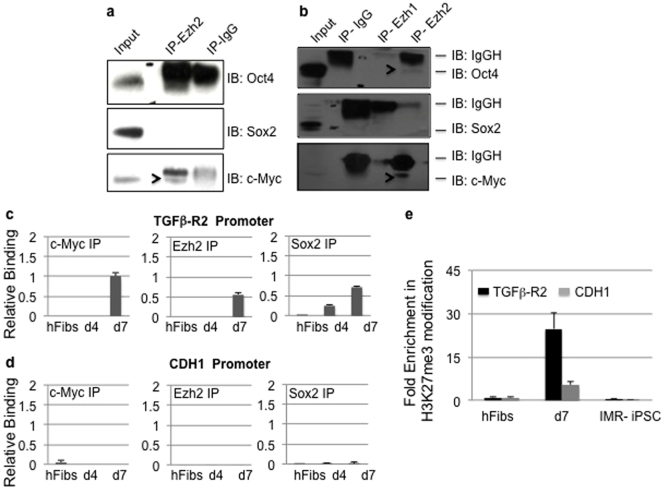
Ezh2 interacts with c-Myc and Sox2 to repress TGF-β receptor and p53 activity during reprogramming. (a) Cell extracts from hFibs transduced with OSKM (day 7) were subjected to immunoprecipitation using Ezh2 or control IgG followed by western blotting for Oct4, Sox2 and c-Myc. Uncropped/Full blots images are provided in Supplementary Fig. 8 a–c (b) Cell extracts from H9-hESC were subjected to immunoprecipitation using Ezh1, Ezh2 or control antibodies followed by immunoblotting for indicated antibodies. Uncropped/Full blots images are provided in Supplementary Fig. 8 d–f. (c–d) Chromatin immunoprecipitation for Sox2, c-Myc and Ezh2 were performed from hFibs, OSKM transduced hFibs at day 4 and 7 days followed by quantitative PCR amplification of indicated gene promoters. (e) Chromatin IP for H3K27me3 was performed in hFibs, OSKM transduced hFibs at day 7 and iPSC.

To dissect the molecular mechanism by which Ezh2 controls iPSC generation, we studied its interaction with pluripotency factors. Protein lysates from OSKM transduced hFibs were collected and subjected to Ezh2 immunoprecipitation [IP]. Western blot analysis followed by Ezh2 IP showed the presence of c-Myc but not Oct4 and Sox2 (Fig. 5a). Such interactions were not seen in control IP IgG reactions indicating the specificity of the immunoprecipitation (Fig. 5a). Next we investigated if Ezh2 associates with OSM factors in pluripotent cells. To address this we performed immunoprecipitation using control, Ezh1 or Ezh2 antibodies using H9 hESC cell lysate. Our results displayed no interaction between Ezh2 and Sox2, however there was weak but detectable amount of Oct4 and c-Myc immunoprecipitated with Ezh2 in pluripotent cells (Fig. 5b). These results suggest that Ezh2 interacts with c-Myc in human fibroblasts undergoing reprogramming and in pluripotent cells.

Because Ezh2 trimethylates lysine 27 on histone3 and transcriptionally represses gene expression, we tested if OSM factors forms a complex with Ezh2 on the TGF-βR2 promoter. We did not observe binding of Oct4 but surprisingly Sox2 was recruited on the TGF-βR2 promoter in day 4 cultures. Sox2 binding was independent of c-Myc-Ezh2 occupancy, which was noticed exclusive at day7 of reprogramming. This result indicates that Sox2 precedes c-Myc and Ezh2 binding on TGF-βR2 locus (Fig. 5c). Amplification of control CDH1 from OSM and Ezh2 immunoprecipitates did not reveal Ezh2 associated repressive complex on the CDH1 promoter at any time point studied (Fig. 5d). Assessment of histone modification status confirmed the presence of H3K27me3 modification on TGF-βR2 but not on CDH1 promoter at day 7 of reprogramming (Fig. 5e). Heavy enrichment of K27me3 mark coincided with downregulation of receptor activity in early time course of reprogramming (Fig. 3). Together, our results demonstrate that Ezh2 transcriptionally represses TGF-βR2 promoter and suppress EMT.

### Ezh2 mediated H3K27me3 activity regulates miR-27a expression during the early phase of reprogramming

**Figure 6:**
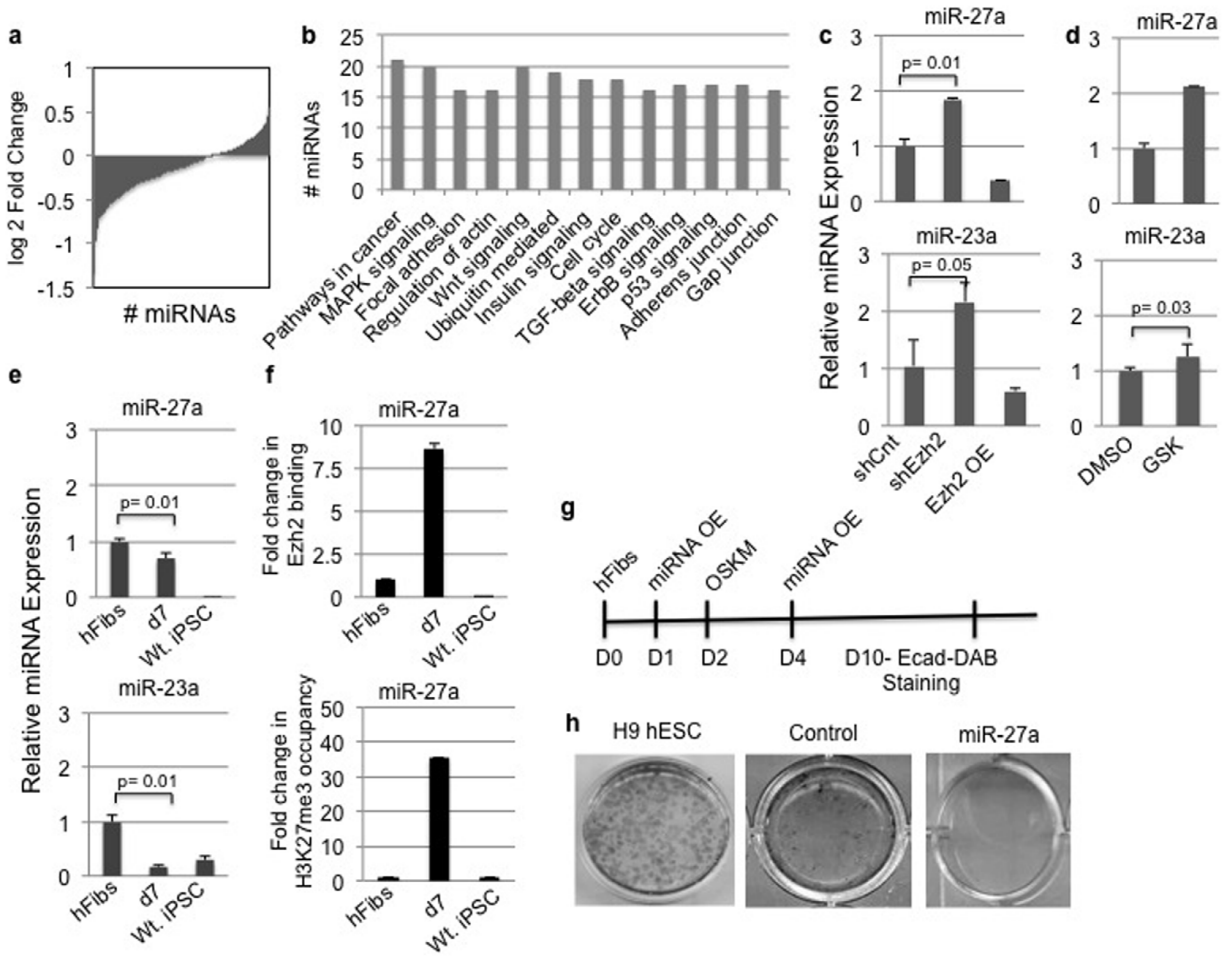
Ezh2 regulates miR-27a expression during human fibroblasts reprogramming. (a) Differentially expressed miRNAs in sh-Ezh2 hFibs calculated by Edge R and DESeq programmes. (b) Histogram depicting the number of miRNA regulating indicated pathways in shEzh2 hFibs. (c) qPCR results of indicated miRNA expression levels in shCnt, shEzh2 and Ezh2 OE hFibs. (d) qPCR results of indicated miRNA expression levels in DMSO control and GSK treated hFibs. (e) Expression profile of indicated miRNA in hFibs, OSKM transduced hFibs on day7 and iPSCs. (f) Chromatin IP for Ezh2 and H3K27me3 was performed in hFbs, OSKM transduced hFibs at day7 and iPSC. Two Kilobase upstream sequence of miR27a was amplified from immunoprecipitated material. (g) Schematic representation of miR-27 plasmid overexpression and OSKM transduction. E-cadherin DAB staining was performed 10 days post introduction of reprogramming factors. (h) E-cad DAB staining in H9 hESC, control and miR-27a overexpressing hFibs.

MicroRNAs play crucial roles in MET of somatic reprogramming and are extensively regulated by PRC2 complex members^31^,^32^. Therefore, we reasoned that depletion of Ezh2 in hFibs Ezh2 might alter miRNA expression thereby influencing the reprogramming process. In order to gain further insights into this we performed small RNA deep sequencing analysis, using biological replicates of shCnt and shEzh2 transduced hFibs. The Pearson coefficient of R> 0.96 shown in Supplementary Fig. 5a–c demonstrated the strong correlation between the biological duplicates. The statistical significance of miRNA expression and fold change was calculated using DESeq and EdgeR (Fig. 6a and Supplementary Table 1 and 2) programmes. miRNAs with p <0.05 in both DESeq and EdgeR analysis were considered for pathway analysis using the Diana miRPath pathway analysis programme. This interrogation revealed a list of signaling pathways that changed significantly upon Ezh2 depletion (Supplementary Table 3). Specifically, genes implicated in cell cycle, p53 and TGF-β signaling were targeted by miRNAs upon Ezh2 depletion (Fig. 6b). This is of particular interest since representative pathways are strongly implicated in pluripotent cells or in iPSC generation (Fig. 6b) and were upregulated upon depletion of Ezh2 in our studies.

Further analysis revealed widespread differences in the abundance of several microRNAs including those that have been previously associated with regulating EMT and MET (Supplementary Fig. 5d). Among those identified miR-23a and miR-27a that belong to miR-23 cluster were overrepresented following Ezh2 knockdown (Supplementary Fig. 5d). Interestingly miR-23a and miR-27a are known to promote EMT in cancers^33^,^34^. Since Ezh2 depletion upregulated TGF-β signaling thereby activating pro-EMT genes (Snail and Twist), it was intriguing to study if Ezh2 regulated the pro-EMT miR-23 cluster directly. Taqman qRT-PCR assays demonstrated increased expression of miR-27a and miR-23a in Ezh2-deficient hFibs confirming our sequencing data (Fig. 6c Upper and Lower panel). If differential expression of miR-23a and miR-27a is directly linked to the knockdown of Ezh2, then overexpression should reverse this trend. As expected, overexpression of Ezh2 reversed the transcript profile of miR-27a and miR-23a (Fig. 6c Upper and Lower panel).

To address the differential activity of depletion of Ezh2 vs inhibition of H3K27me3 activity on expression of miR-23a and miR-27a miRNAs, we performed qPCRs in DMSO and GSK treated cells. Our results indicate enhanced expression of miR-23a and miR-27a in GSK treated cells (Fig. 6d Upper and Lower panel). These results are consistent with our findings demonstrating that the miR-23 cluster is regulated by H3K27me3 activity of Ezh2 in human fibroblasts.

To verify if miR-23a and miR-27a miRNAs are suppressed during the initiation of iPSC generation, we studied their expression pattern during reprogramming. Specifically, we focused on the day 7 timepoint due to molecular changes in Ezh2 expression and initiation of MET as evidenced by emergence of epithelial cells in culture. We detected reduced expression of miR-27a and miR-23a at day 7, which remained downregulated in iPSC cells (Fig. 6e Upper and Lower panel). Downregulation of miR-23a and miR-27a is in alignment with suppression of EMT and acquisition of an epithelial state in iPSC generation. To further confirm that Ezh2 regulates miR-23a and miR-27a expression in reprogramming, we monitored Ezh2 binding and enrichment of H3K27me3 marks on the miR-23 locus in hFibs at day 7 and Wt. iPSCs. Our results demonstrated significant enrichment of Ezh2 and H3K27me3 marks on the miR-23a locus on day 7 of reprogramming compared to hFibs and iPSC cells (Fig. 6f). Collectively, our results demonstrate that the H3K27 activity of Ezh2 overcomes EMT by transcriptional repression of pro-EMT miR-23a and miR-27a during the reprogramming process.

To test the functional role of Ezh2 regulated miR-27a in cellular reprogramming, miR-27a was overexpressed in human fibroblasts (Supplementary Fig. 5e). RT-PCR analysis indicated a four-fold increase in miR27a levels in miR-27a overexpressed hFibs compared to control or untransfected hFib (Supplementary Fig. 5f). We also observed increased expression of Snail and reduced levels of CDH1 in miR-27a overexpressing cells (Supplementary Fig. 5f) compared to controls, indicative of alleviated pro-EMT signaling. Next we directly assayed the role of miR-27a in initiation of reprogramming. Control or miR27a transfected cultures were transduced with OSKM and stained with E-cadherin antibody as shown in the schema (Fig. 6g). Phenotypic analysis combined with E-cadherin staining indicated emergence of E-cadherin positive cells with an epithelial morphology in control cultures while no such changes were noticed in miR-27a overexpressing cultures (Fig 6h). Molecular analysis using transcript profiling confirmed enhanced expression of miR-27a and Snail combined with diminished CDH1 transcripts in miR27a overexpressing reprogramming cultures compared to control (Supplementary Fig. 5g). These results demonstrate that increased miR-27a expression favors the EMT signaling during initiation of reprogramming and acts as an inhibitory microRNA. Taken together our findings confirm that Ezh2 mediated H3K27 activity negatively regulates miR-27a in favor of a mesenchymal to epithelial transition.

## Discussion

Maintenance of cellular identity is subjected to stringent regulatory mechanisms and safeguarded at multiple checkpoints. However, the successful generation of iPSCs has demonstrated that such strict integrity can be altered^1^,^2^,^3^. Recently, detailed molecular characterization of the reprogramming process has revealed distinct steps/waves involved in successful iPSC generation^27^. Among them, activation of mesenchymal to epithelial transition and inhibition of p53-mediated cell senescence are immediate responses following overexpression of OSKM^7^,^35^,^36^. Therefore, to initiate reprogramming, the ectopically expressed pluripotency factors and chromatin modulators must tackle the inhibitory pro-EMT signaling and suppress the stress-response induced by fibroblast via p53, p21 and Ink4/Arf pathways. Global occupancy of these factors revealed similar number of targets of OSK in both the waves, while Myc targeted genes were biased and mostly unregulated in the first wave^8^.

**Figure 7:**
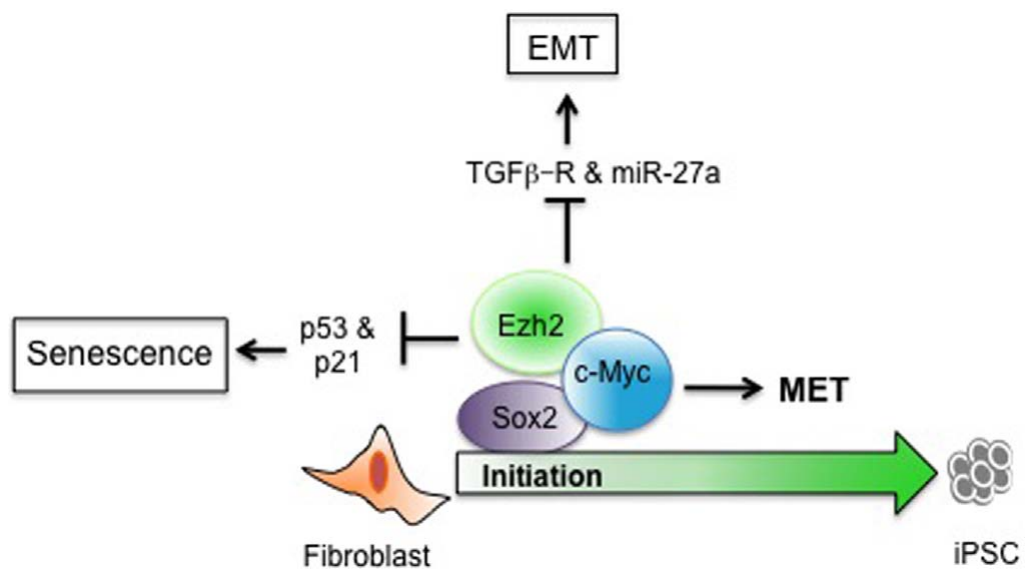
Proposed model by which Ezh2 regulates initiation events in favor of fibroblast transition towards pluripotent state.

Multiple groups have documented the critical role of the chromatin regulator Ezh2 in mouse and human fibroblast reprogramming^12^,^23^,^24^, however the exact mechanism by which Ezh2 alters the epigenome is not clear and therefore an area of great interest. In this regard a recent study by Ding et al^23^ documented that Ezh2 regulates iPSC generation in part by repressing the Ink4A/Arf locus. Our results are in agreement with Ding et al.'s observation wherein depletion of Ezh2 resulted in impaired iPSC generation by induction of p53/ARF pathways [Supplementary Fig. 2]. In the current study, we demonstrate an additional role for Ezh2 in human fibroblast reprogramming and provide mechanistic details by which c-Myc and Ezh2 restrain the somatic program and promote iPSC generation. Enhanced expression of PRC2 throughout the reprogramming process was of particular interest since it provided us with the hint regarding the contributions that repressive H3K27me3 might have in distinct phases of iPSC generation. MET of fibroblast, a key step in reprogramming is subjected to strict intrinsic barriers pro-EMT signaling posed by fibroblast specific transcription factors such as Twist and Snail. Experiments using a small molecule inhibitor of H3K27me3 allowed us to identify the epigenetic basis of regulation of EMT signaling in favor of MET during initiation of reprogramming. Our combined results from Ezh2 perturbation and early fibroblast reprogramming studies demonstrate a negative correlation between Ezh2 and TGF-β receptors and its downstream signaling in reprogramming time courses. Soufi et al., (2012)^8^ have demonstrated binding of OSM to the TGF-β promoter during the early hours of reprogramming. Consistent with this finding, we provide evidence of H3K27me3 marks along with localization of an Ezh2, c-Myc and Sox2 repressor complex on the TGF-βR2 promoter. Further we demonstrate that Ezh2 modulates Snail expression via TGF-β signaling. Enhanced Snail expression in shEzh2 hFibs seems to repress CDH1 transcription. This assumption is supported by previous studies where Snail is shown to bind to CDH1 promoter and repress its transcription^37^. Our experiment demonstrating the rescue of MET defects upon inhibition of TGF-β signaling convincingly demonstrates the role of Ezh2 in inhibiting the fibroblast specific programme. Overall our work provides the mechanism for the initial action of c-Myc and Ezh2 to target chromatin sites to disrupt the existing somatic programme and to facilitate the somatic exit. Most importantly we identified previously unreported microRNAs, miR-23a and miR-27a, as barriers in human iPS generation. Binding of Ezh2 and localization of H3K27me3 marks on miR-23a locus further supports our claim that H3K27me3 activity is critical to negate miRNA mediated pro-EMT signaling. In conclusion, our data demonstrates that Ezh2 favors iPSC generation by directly repressing fibroblasts specific genes and also by miRNA mediated trans-regulatory mechanisms to keep these signaling pathways under control (Fig. 7).

Our observations are consistent with Onder et al.^12^; Buganim et al.^25^; Ding et al.^23^ studies demonstrating inactivation of Ezh2 inhibited cellular reprogramming. In contrast Fragola et al.^38^ did not observe impaired reprogramming upon genetic inactivation of Ezh2 and its catalytic activity. In addition they fail to notice the cellular senescence defects in Ezh2 deficient MEFs. Such observations may have been unnoticed due to lack of efficient Ezh2 depletion in early phase of reprogramming. As such we demonstrated the requirement of H3K27me3 activity in the inhibition of pro-EMT signaling. In the future, it would be important to determine how multiple epigenetic regulators collaborate with OSKM and work in concert to establish the epigenomic networks to govern differential patterns of permissive and non-permissiveness on gene loci during iPSC generation.

## Experimental Procedures

### Antibodies and Inhibitors

We used antibodies specific to the Ezh2 (Millipore #17-662 and #CS203195), c-Myc (9E10) (abcam ab32), Sox2 (abcam ab59776), H3K27me3 (abcam ab24684), Oct3/4 (c-10) (Santa Cruz Sc5279), Nanog (abcam ab21624),) Pan H3 (ab1791), GAPDH (abcam ab9485), CDKN2A/p14ARF (abcam ab470), TGF-β RI (C-4) (Santa Cruz Sc-17791), TGF-β RII Antibody (C-16) (Santa Cruz Sc220), Normal Rabbit IgG (Millipore #12-370), Normal Mouse IgG (Millipore #12-371), p21 (F-5) (Santa Cruz sc-6246), Phospho-Smad2 (Ser465/467) (138D4) (Cell Signaling 3108), Smad2 (D43B4) (Cell Signaling 5339), E-Cadherin (Abcam#1416). TGF-β inhibitor SB431542 was purchased from Stemgent (04-0010). H3K27me3 inhibitor GSK-126 was purchased from Xcesbio.

### Cell Culture

Fetal human dermal fibroblasts were purchased from Sciencell and maintained in fibroblast medium (DMEM supplemented with 10% FBS, L-glutamine, NEAA). HEK-293, 293-LX and 293-Ampho cells were cultured in DMEM medium supplemented with 10% FBS, L-glutamine, NEAA and sodium pyruvate. IMR-iPSC generated from fetal lung fibroblast and H9 hESCs were purchased from WiCell and maintained in mTESR media on hESC grade matrigel. For generation of stable cell lines expressing Ezh2 shRNA (shEzh2), 1 μg of vector DNA was transfected into 293 cells using lipofectamine 2000 reagent (Invitrogen). Forty-eight hours post transfections clones were selected for stable integration of plasmids using 1.5 μg/ml puromycin.

### Viruses

Sendai virus encoding human Oct4, Sox2, c-Myc and Klf4 was purchased from Invitrogen (Cytotune- A1378001). Lentiviral vectors pLKO.1 NTS Control shRNA (SHC016), pLKO.1 EZh2-74 shRNA (TRC000010474), pLKO.1 EZh2-75 shRNA (TRC000010475), were purchased from Sigma. These vectors were co-transfected with psPAX2, pMDG2 in 293-LX packaging cell line using lipofectamine LTX (Invitrogen). Viral supernatants were harvested 48 h post transfection and concentrated using Amicon filters (Millipore). Human fibroblasts were transduced with control and Ezh2 shRNA at MOI#3 in presence of 8-ng/ml polybrene. shEzh2-75 referred as shEzh2 in the manuscript was used in almost all the experiments unless otherwise mentioned.

Retroviral MSCV-Ezh2 vector was purchased from Addgene. This vector was transfected in AmphoPack-293 cell line (Clontech). Viral supernatants were harvested 48 h post transfection. 2 ml of viral supernatant was used to transduce hFibs.

### Transductions

For Ezh2 depletion or overexpression experiments, 20,000 hFibs were transduced. Forty-eight hours-post viral infection cells were washed off with 1X PBS and DMEM and supplemented with fresh complete medim. Transduced fibs were cultured for another 48 hrs and analyzed in various experiments including PCRs, western blotting and senescence assays. In case of TGF-β signaling inhibition experiments, 2 μM SB431542 was added to the hFibs post 48hs of transduction with shRNAs. Cells were cultured for additional 48 h prior to analysis.

### Inhibitor treatment

Equal number (20,000) of hFibs was seeded on 12- well dishes. Twenty-four hours post seeding, H3K27me3 inhibitor GSK was added at 0.5 μM concentration. Treated cells were analyzed by RT- PCRs four days post treatment.

### Induction of reprogramming

For reprogramming experiments 50,000 hFibs were seeded on matrigel coated wells. For shRNA mediated depletion experiments hFibs were pre-transduced with NTS-control shRNA referred as shCnt, Ezh2 shRNA referred as shEzh2 and untransduced referred as Wt hFibs. Twenty-four hours post shRNA transduction hFibs were washed with knockout DMEM and transduced with OSKM Sendai (Cytotune kit Invitrogen) in complete hFib medium. OSKM transduced hFibs were switched to iPSC medium [iPSC medium containing Knockout DMEM, KOSR, NEAA, L-Glut, β-Mercaptoethanol and bFGF] after 48 hrs. In case of TGF-β signaling inhibition experiments 2 μM SB431542 was added to the iPSC medium. Colonies were picked manually three weeks post reprogramming to establish iPSC lines. Later iPSC cultures were maintained in E8 and/or mTESR medium. In case of inhibition of H3K27me3 experiments 0.5 μM GSK was added to the iPSC medium.

### Cloning of miR-27a

miR-27 was amplified from human genomic DNA with primers containing the appropriate restriction enzyme sites (Fw mir-27a-AgeI- 5′-GATC***ACCGGT***GTCACAAATCACATTGCCAGG-3′; Bw-mir27a-HindIII- 5′-GATC***AAGCTT***TCAGTAGGCACGGGAGGCAGA-3′). The amplicon containing the pre-miR-27 flanking sequences and was cloned down stream to CB promoter with CMV enhancer in the plasmid containing AAV inverted terminal repeats.

### Transfection of miR-27a

Following manufacturers instructions magnetic assisted transfection (Magnetofection Kit) of control or miR-27a was performed in human dermal fibroblast instructions (Ozbiosciences Cat # NM51000). Transfection efficiency of 40% - 50% was standardized GFP expression using control pAAV-Vector.

### Small RNA library Preparation and Sequencing

Small RNA libraries were prepared using the Illumina TruSeq Small RNA kit as described by the manufacturer (Illumina). 250 ng of total RNA was used from each sample for the library preparation. 5′ and 3′ Small RNA adaptors were ligated to the RNA and the ligated products were reverse transcribed using SuperScript II reverse transcriptase (Invitrogen). The reverse transcribed products were then amplified by polymerase chain reaction and resolved on 8% polyacrylamide gel. Bands corresponding to 140 – 160 nucleotides were gel eluted. The size and integrity of each library was verified using the Bioanalyzer. The libraries were sequenced on an Illumina HiSeq 1000.

### Computational Analysis

Reads were aligned to both the draft assembly of the *Human genome hg19, (UCSC*) and the known *human* miRNAs from miRBase using Bowtie-0.12.1^39^. Adapter sequence trimming was performed using inhouse perl script. Approximately 38 million raw reads of 18-24nt lengths from shCnt and 19 million reads from ShEzh2 were obtained. Using no mismatch criteria 68–75% reads from shCnt and 53–60% reads from sh-Ezh2 hFibs mapped to human genome. Of which 90–93% of mapped reads from shCnt and 68–70% mapped reads from shEzh2 were aligned to human miRNAs from miRbase. (Supplementary Table 1). Reads that mapped to miRNAs were normalized to the total number of mapped reads per million reads (Reads per million). Fold change enrichment for the miRNAs in shEzh2 was calculated as the ratio between the normalized reads for each miRNA from the shEzh2 to the normalized reads for that miRNA in shCnt sampales. DeSeq and EdgeR programmes were used to identify the differentially expressed miRNAs. miRNAs with p value less than 0.05 have been reported. In DeSeq, false discovery rate method was used to calculate the adjusted p value and for EdgeR we used bonferroni correction for calculating adjusted P values^40^. To identify the altered signaling pathways in shCnt Vs. shEzh2 samples, miRNAs with p value less than 0.05 were used to run online Diana miRPath v.2.0 tools^41^.

### PCRs, RT-PCRs and miRNA Validations

Total RNA was isolated using Trizol reagent (Invitrogen) as per manufactures instruction. RNA was then subjected to cDNA synthesis using superscript III (Invitrogen). Semi-quantitative PCR reactions were performed using 2X PCR Master Mix (Fermentas). Products were resolved on 1.2% agarose gels. Quantitative PCRs were performed using Platinum SYBR Green –UDP mix (Invitrogen). To calculate the relative gene expression, GAPDH mRNA was amplified as internal control. Primer sequences are provided in supplementary table 2 (Supplementary Table 4).

For validation of miRNAs, 50 ng of total RNA was reverse transcribed using TaqMan MicroRNA Reverse Transcription Kit (Applied Biosystems). cDNA was synthesized by using specific primers for, miR -27a (002445), miR- 23a (000399) or control U6 SnoRNA (001973) and amplified according to manufacturers instructions (Applied Biosystems). Subsequently Taqman assays against indicated microRNAs were used for quantification on an Applied Biosystem 7900HT Fast Real-Time PCR system. The control SnoRNA was used for normalization.

### Live Staining

Live staining was performed using sterile Tra-1-60 antibody pre-conjugated with Alexa Fluor 488 goat anti-mouse Ig-M (Molecular Probes, Invitrogen) at room temperature. Reprogrammed colonies were washed once with iPSC medium and incubated with Tra -1-60-Alexa 488 for 30 minutes at room temperature. Cultures were then washed twice to remove unbound antibody. Cells were visualized using the Olympus fluorescence microscope and images were captured using the Q Capture software.

**DAB staining.** For DAB staining reprogramming culture IMR-90 iPSCs or H9-hESC were fixed with 4% paraformaldehyde for 15 min at room temperature. After three washes with 1XPBS, the fixed cultures were incubated in 1X PBS containing 1% bovine serum albumin (Invitrogen), and 0.25% Triton X-100 for 30 min at room temperature. Cultures were washed with 1X PBS and were incubated with Primary antibody Tra 1-60 (1:100) (Santacruz) or E cadherin (1:100)(Abcam) for 2 hours. Cultures were washed three times with 1X PBS to remove unbound antibody and incubated with Goat anti mouse horseradish peroxidase (Biorad, 1:500) for one hour. Staining was developed with the Vector labs DAB and visualized by an Olympus microscope.

### Senescence Assay

20,000 hFibs were transduced with shCnt, shEzh2 or Ezh2 OE encoding viruses. Cells were washed with PBS, fixed, and stained using the Senescence Kit (Sigma). Stained cells were visualized using a Nikon Eclipse TE 2000-S microscope under bright field, and images were acquired using the Q Capture software.

### Immunocytochemistry

hiPSC colonies were fixed in paraformaldehyde and permeabilized in Triton X-100 prior to Oct4 and Nanog staining. Cells were then stained with secondary antibody Alexa Fluor 488 anti- IgG (Molecular Probes). DAPI staining was performed to visualize the nucleus (Molecular probes). Cells were visualized using the Olympus IX81 fluorescence microscope and images were captured with using the Q Capture software.

### Western blotting and Immunoprecipitation

Cell extracts were prepared in RIPA buffer. Equal amount of protein was loaded for western blotting with indicated antibodies. For immunoprecipitation studies, protein A or protein G Dynabeads were coated with 2–3 μg of indicated antibodies. Approximately 400 μg of protein lysate from shCnt or shEzh2 stable lines overexpressing OSKM were subjected to immunoprecipitation. Beads were then washed three times with buffer containing 300 mM KCl and 0.1% (v/v) Nonidet P-40, and proteins were eluted in SDS loading dye and then subjected to western blotting.

### Chromatin Immunoprecipitation

Chromatin IP was performed as described previously^42^. In brief cells were crosslinked using 1% formaldehyde and chromatin was digested in buffer containing 0.1% SDS to obtain fragments of approximately of 400 bp length. Sonicated DNA was subjected to immunoprecipitation using ChIP grade antibodies anti-Ezh2, anti c-Myc, anti Sox2, rabbit IgG and mouse IgG antibodies. Immunoprecipitated DNA was further reverse crosslinked, purified and subjected to qPCR analysis using Platinum Syber Green-UDP mix. To calculate relative binding, we subtracted the signal observed in the control (IgG control) immunoprecipitation experiment from that observed with the specific antibody. Then we divided the resulting difference (IP specific – IP IgG) by the signal observed from 1/50th of the ChIP input material. ChIP data graphs were plotted from absolute values obtained from {(IP Specific- IP IgG)/ Input}. For Supplementary Fig. 3c, the absolute value obtained from one of the technical replicate of shCnt was considered as 100% and shEzh2 was plotted against that. Standard deviations were calculated using three biological replicate for control shRNA and Ezh2 shRNA.

### Statistical Analysis

Results are presented as the mean ± SEM of at least 3 independent experiments unless stated otherwise. Significance levels were determined using 2-tailed Student's **t**-tests where p value is indicated in the figure.

## Author Contributions

S.R. conceptualized the project and designed the experiments. N.D. and R.R. performed experiments on lenti, retrovirus production, shRNA transduction, PCRs, immunostaining, immunoprecipitation and ChIP studies. S.R. and R.R. are responsible for iPSC generation and maintenance of iPSC lines. N.D. and A.K. performed the Ezh2 inhibitor studies. S.C. and J.G.R. cloned miR-27a for overexpression studies. D.P. is responsible for generating small RNA libraries and bioinformatics analysis of small RNA sequencing. S.R. and D.P. wrote the paper, discussed and commented on the manuscript.

## Supplementary Material

Supplementary Information Supplementary Figures
