# Supplementary material for: Ezh2 mediated H3K27me3 activity facilitates somatic transition during human pluripotent reprogramming
Source: Sci Rep. 2015 Feb 4;5:8229. doi: 10.1038/srep08229 (PMC4316165; doi:10.1038/srep08229)

Supplementary Information

Ezh2 mediated H3K27me3 activity facilitates somatic transition during human pluripotent reprogramming

Radhika Rao*[1,2], Narendra Dhele*[1], Sabna Cheemadan[3], Alhad Ketkar[1], Giridhara R. Jayandharan [3], Dasaradhi Palakodeti[1] and Shravanti Rampalli[1]

Supplementary Figure Legends

Supplementary Figure 1: knockdown of Ezh2 in hFibs negatively influences iPSC generation. (a-c) Pluripotency marker expression in H9 hESC and representative iPSC's colonies from hFibs.

Supplementary Figure 2: Depletion Ezh2 induces cell senescence in human fibroblasts. (a) Western blots analysis using anti Ezh2 and GAPDH antibody in HEK-293 control, stable lines expressing shCnt and shEzh2. **(b)** Relative Ezh2 expression in shCnt or shEZh2 transduced hFibs. **(c)** Western blots analysis using a-Ezh2, α-H3K27me3 and α-pan H3 antibodies in hFibs transduced with shCnt and shEzh2. **(d)** Schematic representation for shEzh2 transduction, OSKM infection and iPSC generation from hFibs. **(e)** Total number of Tra1-60 +ve colonies generated 3-weeks post OSKM transduction from 20,000 wt, shCnt or shEzh2hFibs. **(f)** Relative expression of mRNA encoding p21, p53 and p19 in wild type hFibs, hFibs transduced with shCnt or shEzh2. **(g)** Western blotting for indicated proteins including GAPDH control in wild type hFibs, shCnt hFibs, and shEzh2 hFibs. **(h)** {upper panel} Representative bright field images of Wt. hFibs and hFibs transduced with shCnt or shEzh2. {lower panel} β-gal staining indicating cell senescence in shEzh2 transduced hFibs compared to that wild type and shCnt controls. **(i)** Relative expression of Ezh2 in Wt. iPSC and Wt. iPSC transduced with shEzh2. **(j)** Bright field, DAPI, Tra 1-60, and Nanog staining in sh-Ezh2 transduced iPSCs.

Supplementary Figure 3. Altered Ezh2 levels influences the expression of TGF-β and BMP receptors. (a) Relative expression of TGF-β ligands in hFibs transduced with control and Ezh2 shRNA. **(b)** Quantitative PCR for Ezh2 in hFibs transduced

with or without retrovirus expressing Ezh2 transgene. **(c)** Western blot analysis using anti Ezh2, H3K27me3 and Pan Histone 3 antibody in control hFibs or hFibs transduced retrovirus overexpressing Ezh2 transgene (Ezh2 OE). **(d)** Relative expression of mRNA encoding TGF-β receptors in hFibs transduced with retrovirus encoding Ezh2 transgene. **(e)** Chromatin immpunoprecipitation of control antibody Pan-H3 and H3K27Me3 in ShCnt and ShEzh2 transduced hFibs followed by quantitative PCR amplification of TGF-βR2 gene promoter. **(f)** Relative expression of BMP receptors 1and 2 in hFibs transduced with control and Ezh2 shRNA.

Supplementary Figure 4. Inhibition of TGF-β signaling does not improve the reprogramming competency in Ezh2 depleted hFibs. **(a)** Schematic representation for Ezh2 shRNA transduction, OSKM infection and SB treatment during reprogramming from hFibs. **(b)** Total number of E-cadherin positive colonies 10 days post OSKM transduction from wild type hFibs; Ezh2 depleted hFibs with or without TGF-β signaling inhibitor SB431542. **(c)** Relative expression of mRNA encoding Ezh2, TGF-β receptors and E-cadherin in hFibs transduced OSKM, control shRNA expressing hFibs, Ezh2 shRNA expressing hFibs with or without SB431542. **(d)** Bright filed images for fibroblasts transduced with shCnt and shEzh2 with or without TGF-β signaling inhibitor SB431542. **(e)** Relative expression of mRNA encoding p53 and p21 in hFibs transduced OSKM, control shRNA expressing hFibs, Ezh2 shRNA expressing hFibs with or without SB431542.

Supplementary Figure 5. Differentially expressed miRNA in shEzh2 hFibs regulate the TGF-β signaling. **(a-b)** Scatter plot representing the miRNA distribution pattern between the biological replicates of shCnt or shEzh2. R^2 values represent the Pearson co-efficient. **(c)** Scatter plot representing the miRNA distribution pattern between the shCnt and shEzh2 transduced hFibs. **(d)** Relative percent reads mapped for indicated miRNAs in shCnt Vs shEzh2 hFibs. **(e)** Plasmids used for control and miR-27 overexpression in reprogramming assays **(f)** Relative expression of indicated mRNAs in control and miR-27a overexpressing hFibs. **(g)** Relative expression of indicated mRNAs in control and miR-27a reprogramming cultures.

Supplementary Figure 6. Uncropped full length blots for Figure 2a and Figure 2d in the main text.

Supplementary Figure 7. Uncropped full length blots for Figure 3c and Figure 3e in the main text.

Supplementary Figure 8. Uncropped full length blots for Figure 5a and Figure 5b in the main text.

Rao et al; Supplementary Figure 1

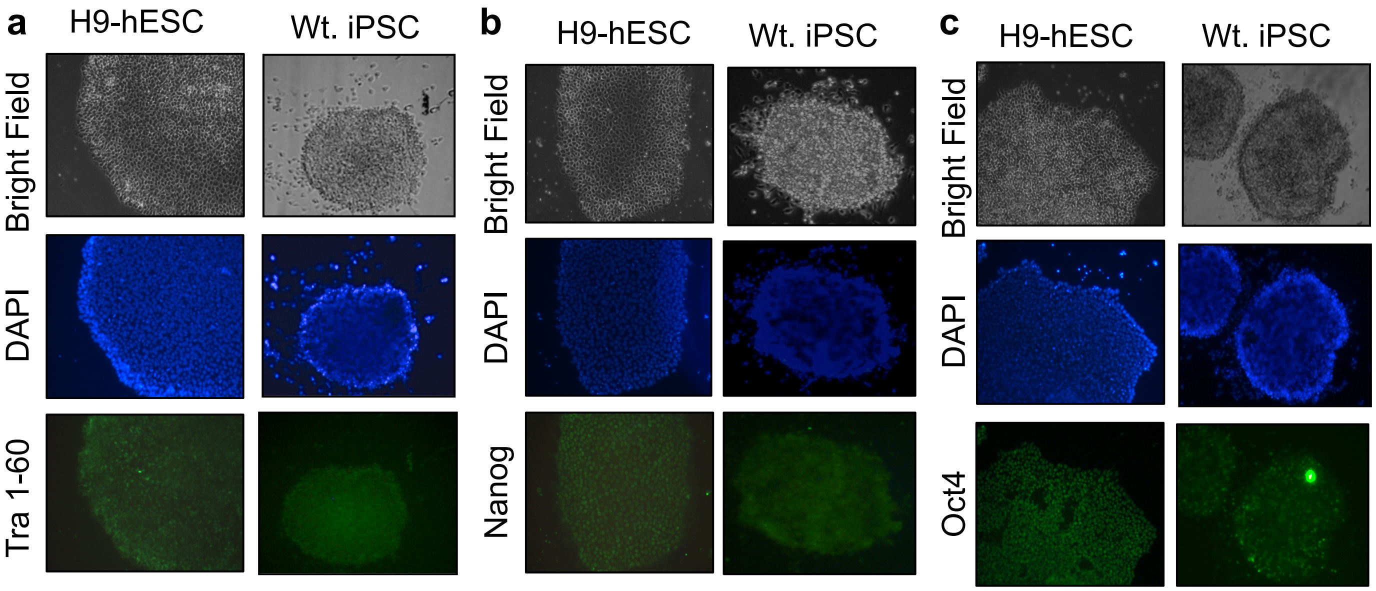

Rao et al; Supplementary Figure 2

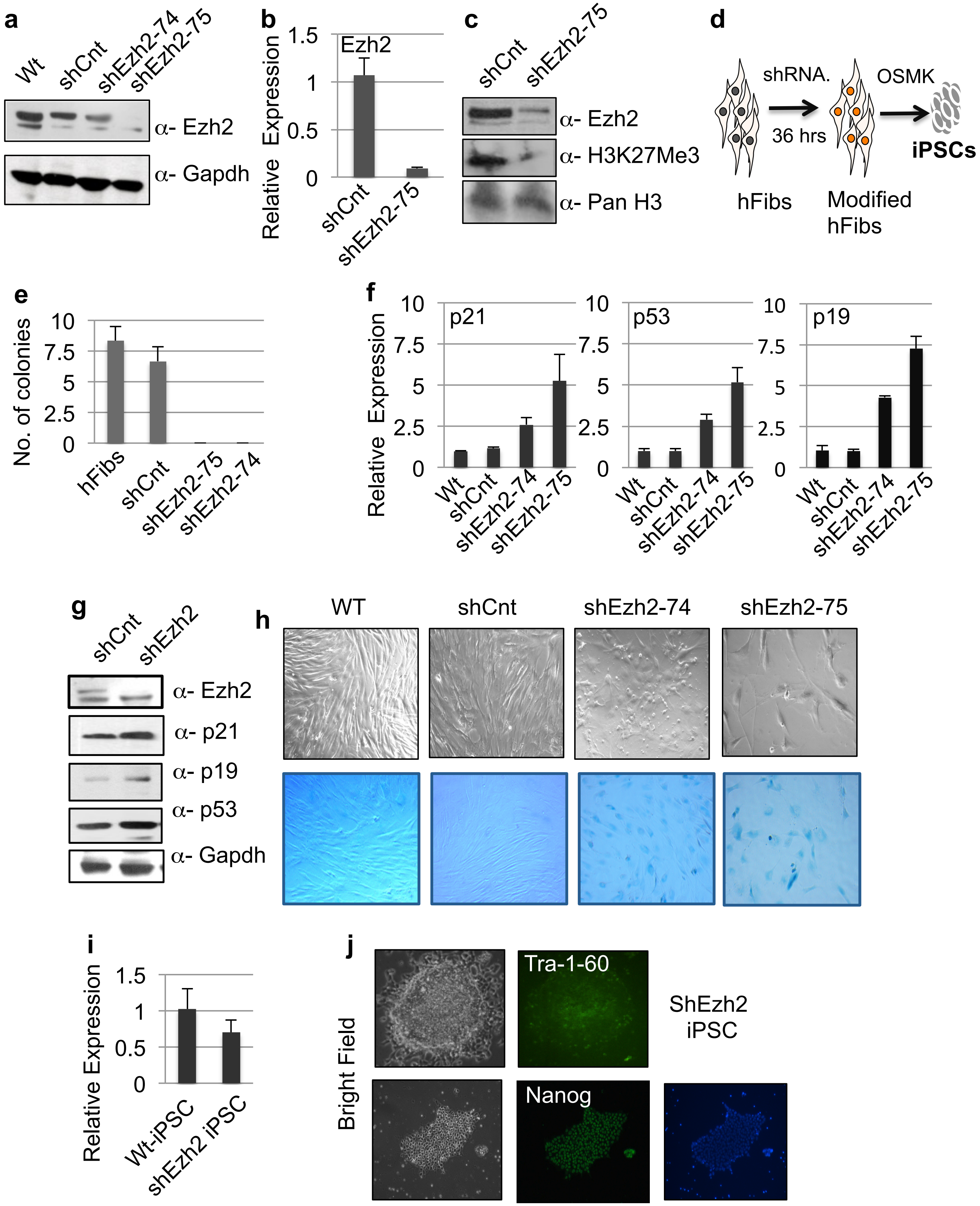

Rao et al; Supplementary Figure 3

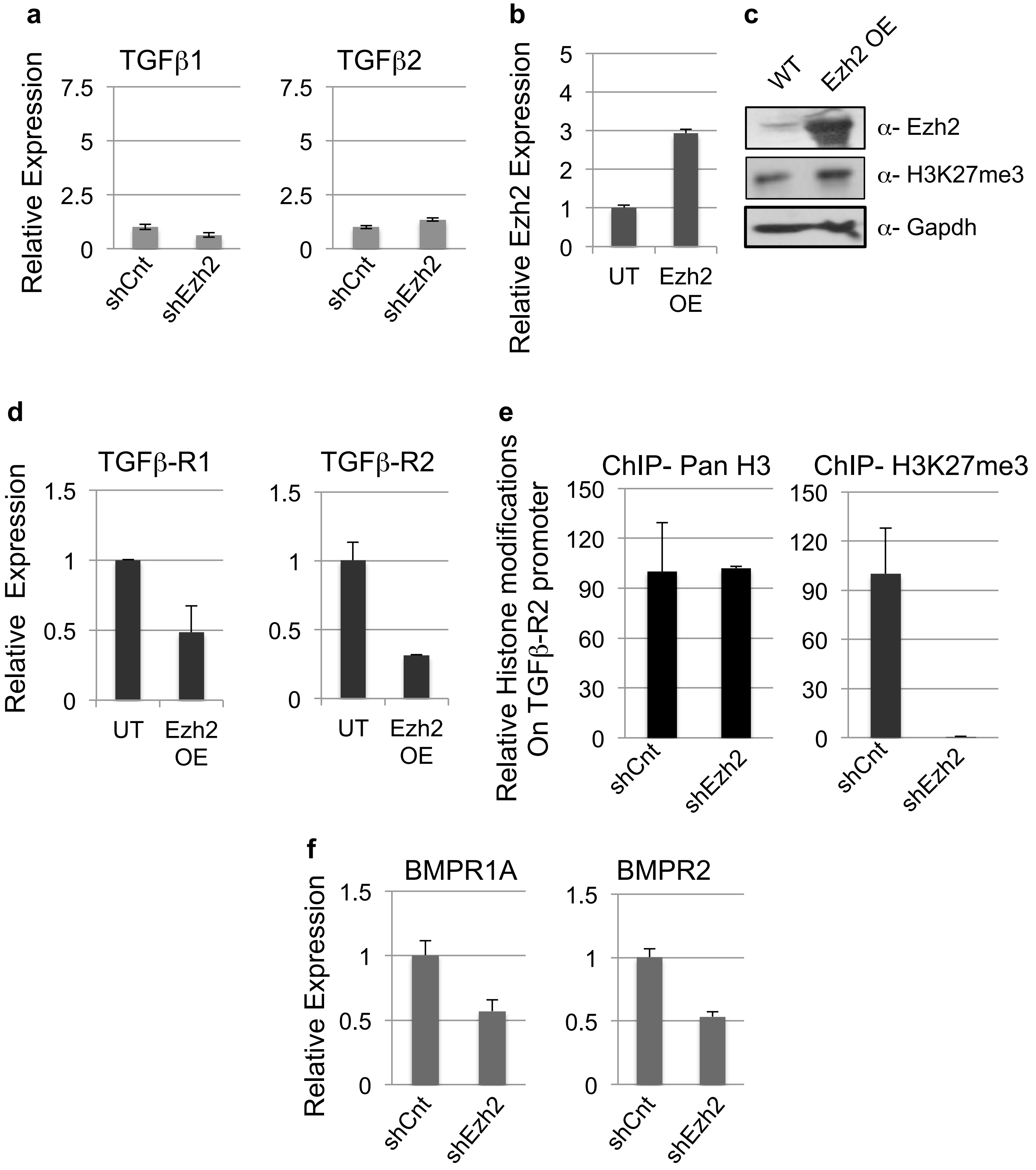

Raoi et al; Supplementary Figure 4

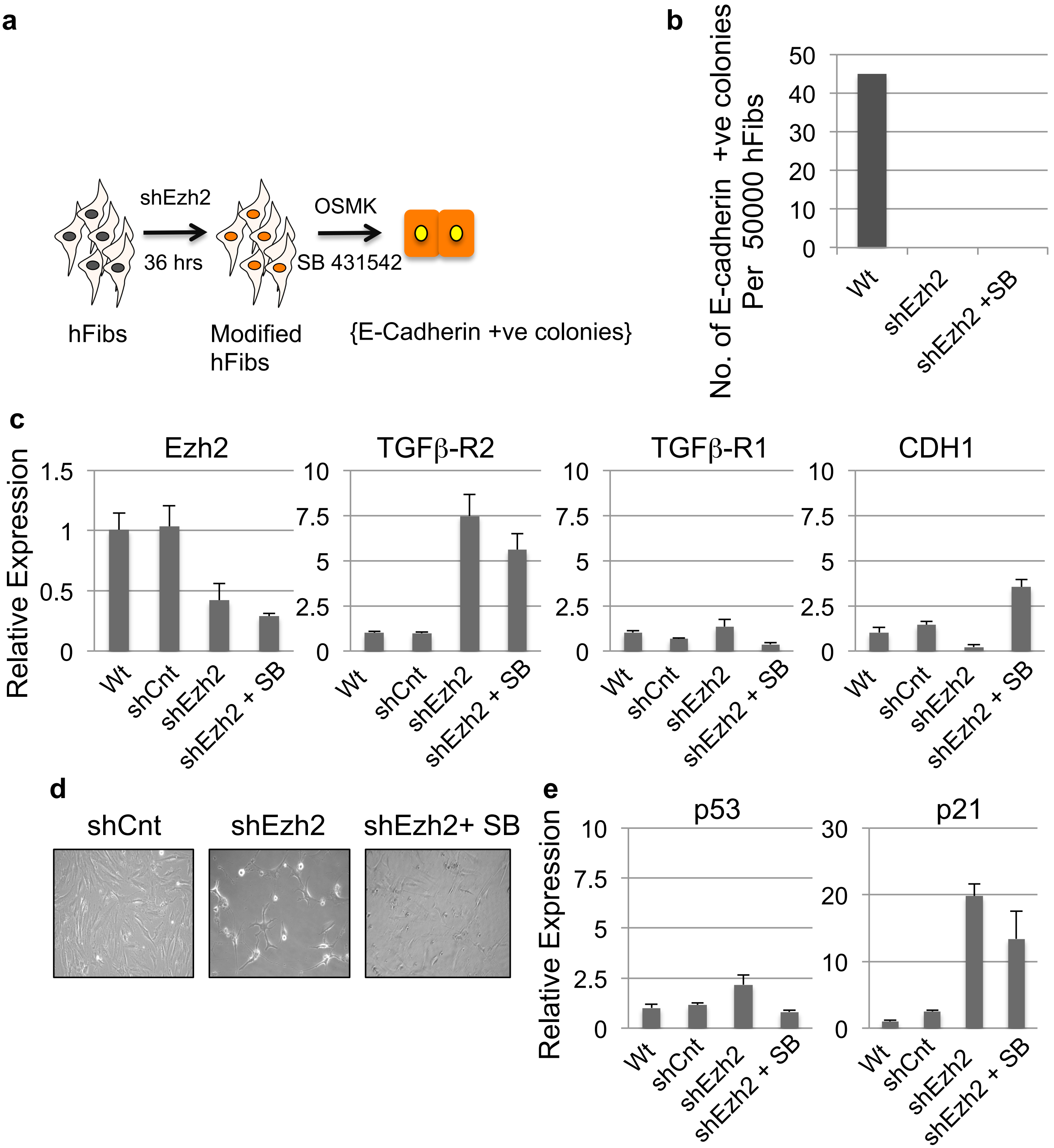

Rao et al; Supplementary Figure 5

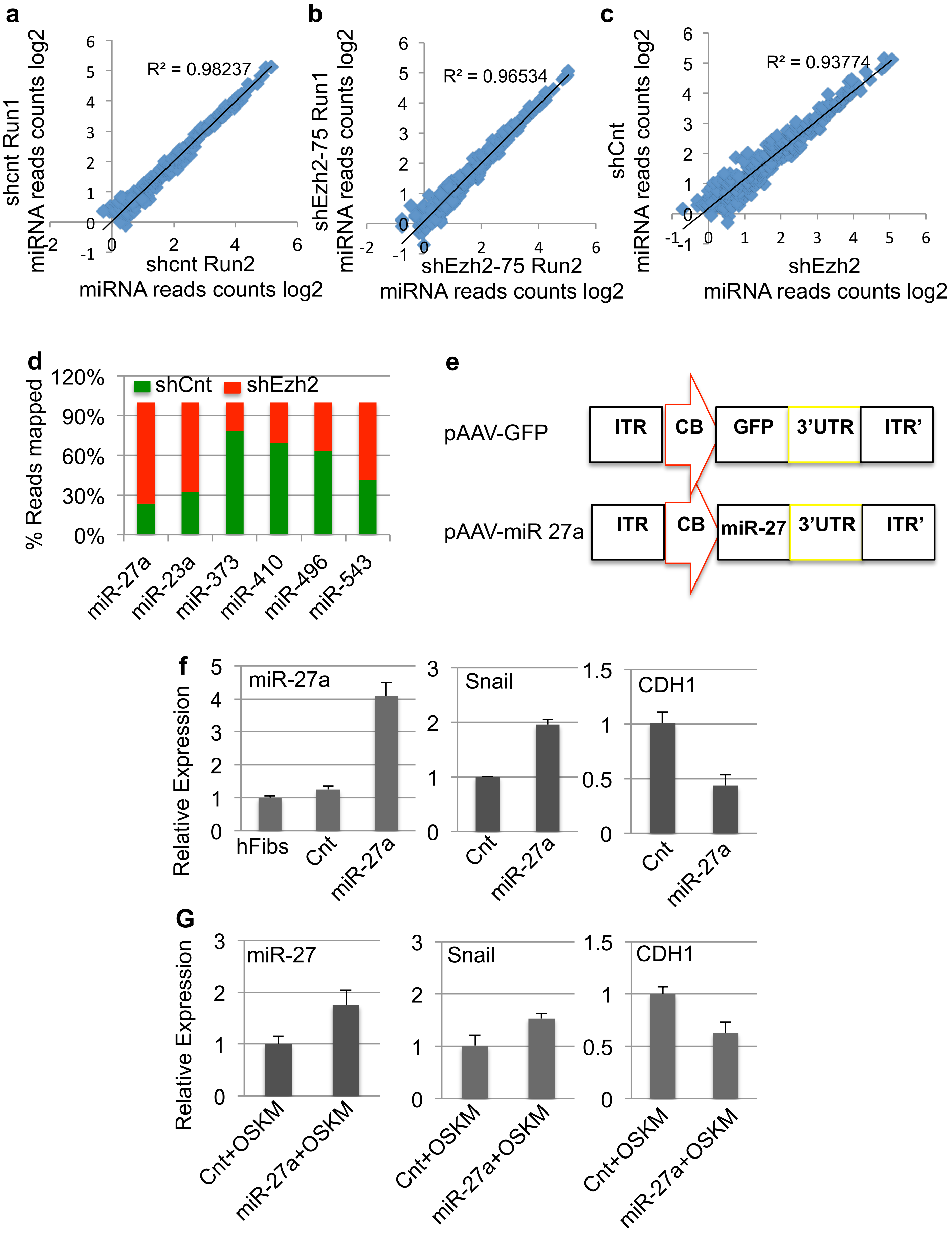

Rao et al; Supplementary Figure 6

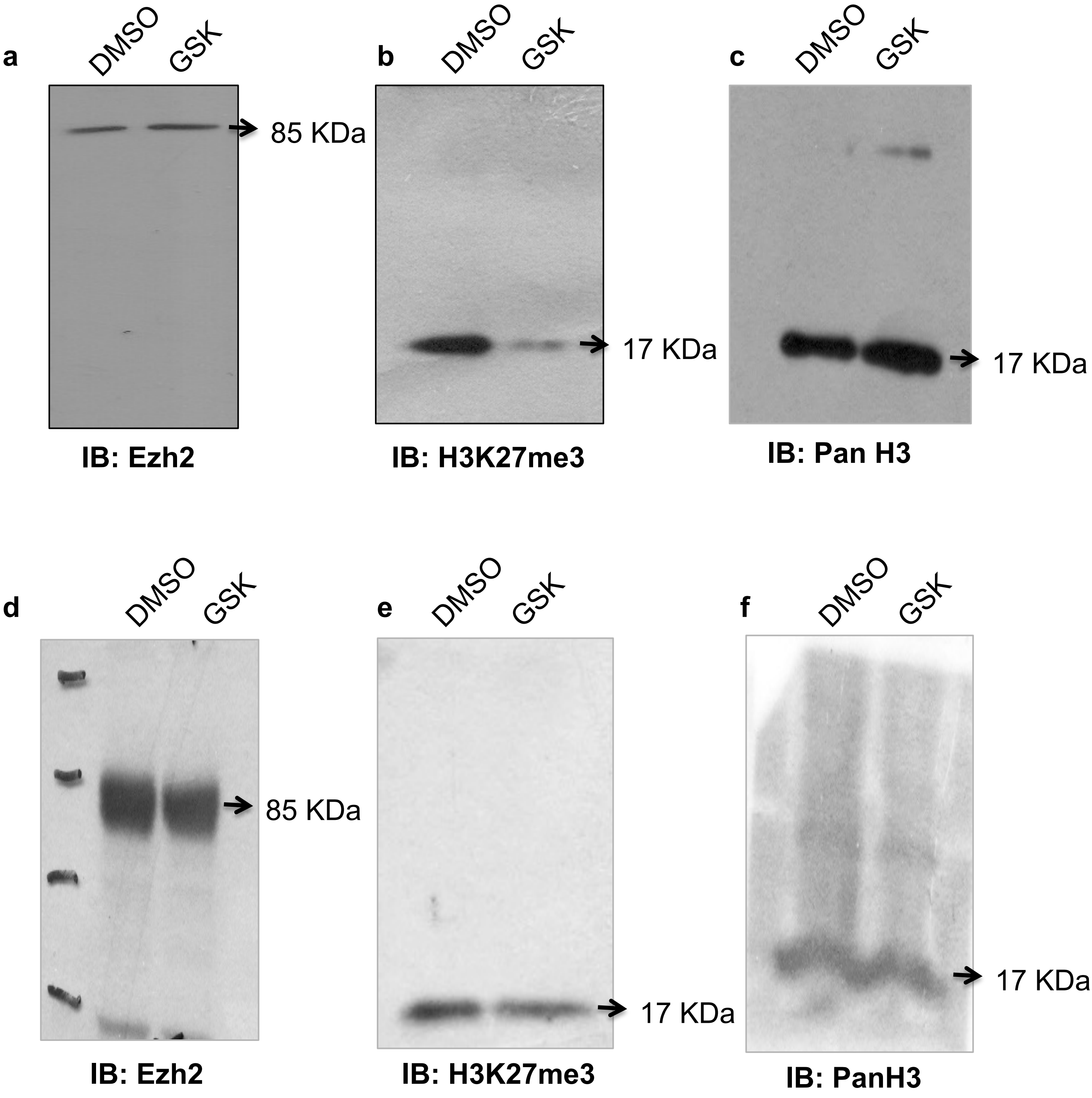

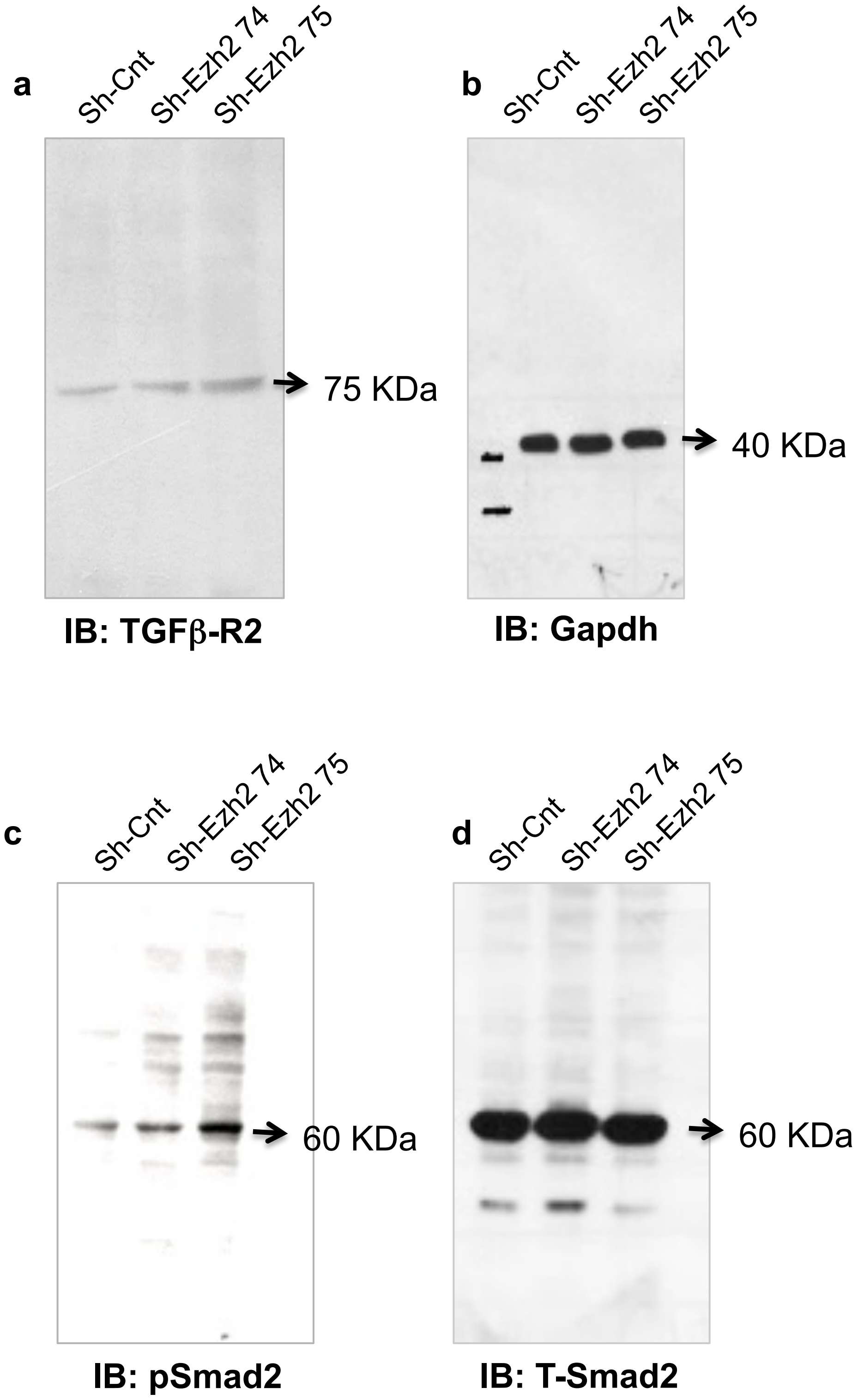
a
Sh-Cnt
Sh-Ezh2 74
Sh-Ezh2 75
75 KDa
IB: TGFβ-R2
b
Sh-Cnt
Sh-Ezh2 74
Sh-Ezh2 75
40 KDa
IB: Gapdh
c
Sh-Cnt
Sh-Ezh2 74
Sh-Ezh2 75
60 KDa
IB: pSmad2
d
Sh-Cnt
Sh-Ezh2 74
Sh-Ezh2 75
60 KDa
IB: T-Smad2

Rao et al; Supplementary Figure 8

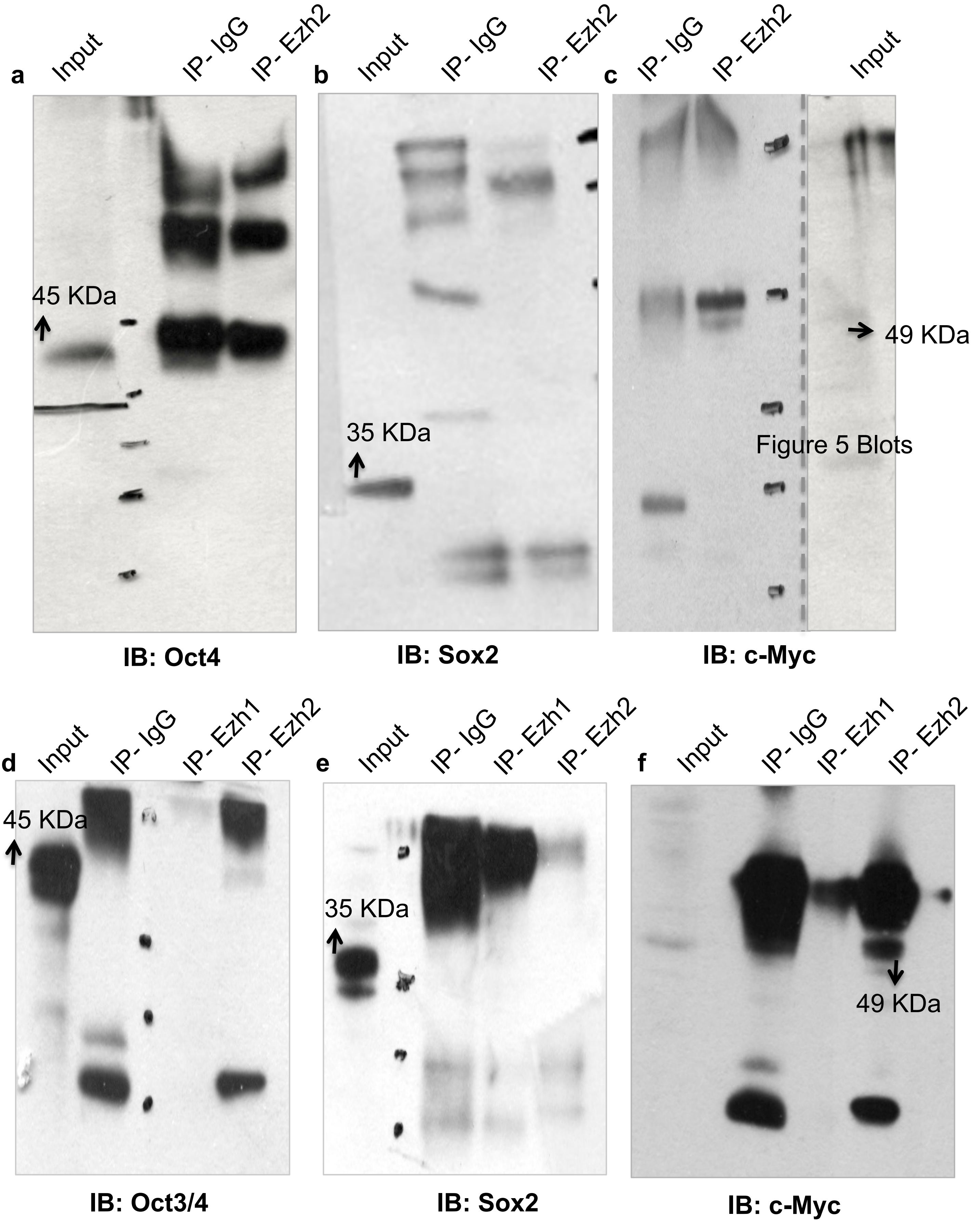

Supplement: Supplementary Information — Supplementary Figures [file srep08229-s1.pdf]
